# Caloric vestibular stimulation has no effect on perceived body size

**DOI:** 10.1038/s41598-019-47897-9

**Published:** 2019-08-06

**Authors:** Hans-Otto Karnath, Isabel Kriechel, Joachim Tesch, Betty J. Mohler, Simone Claire Mölbert

**Affiliations:** 1 Centre of Neurology, Division of Neuropsychology, Hertie-Institute for Clinical Brain Research, University of Tübingen, Tübingen, Germany; 2 Department of Psychology, University of South Carolina, Columbia, SC 29208 USA; 3 Max Planck Institute for Biological Cybernetics, Tübingen, Germany; 4 Technical University Darmstadt, Institute of Sports Science, Darmstadt, Germany; 5 Medical University Hospital Tübingen, Dept. of Psychosomatic Medicine and Psychotherapy, University of Tübingen, Tübingen, Germany

**Keywords:** Perception, Sensory processing

## Abstract

It has been suggested that the vestibular system not only plays a role for our sense of balance and postural control but also might modulate higher-order body representations, such as the perceived shape and size of our body. Recent findings using virtual reality (VR) to realistically manipulate the length of whole extremities of first person biometric avatars under vestibular stimulation did not support this assumption. It has been discussed that these negative findings were due to the availability of visual feedback on the subjects’ virtual arms and legs. The present study tested this hypothesis by excluding the latter information. A newly recruited group of healthy subjects had to adjust the position of blocks in 3D space of a VR scenario such that they had the feeling that they could just touch them with their left/right hand/heel. Caloric vestibular stimulation did not alter perceived size of own extremities. Findings suggest that vestibular signals do not serve to scale the internal representation of (large parts of) our body’s metric properties. This is in obvious contrast to the egocentric representation of our body midline which allows us to perceive and adjust the position of our body with respect to the surroundings. These two qualia appear to belong to different systems of body representation in humans.

## Introduction

Beginning with early work by, e.g., Bonnier^1^ or Schilder^2^ it has been suggested that the vestibular system not only plays a role for our sense of balance and postural control but also contributes to the perception of state and presence of our body relative to the environment. In this line, it has been discussed that the multisensory (vestibular) cortex in humans^3,4^ may serve as a convergence zone of various sensory input, involved in perceiving shape and size of the body and in generating bodily self-representation^1,2,5–8^. In line with these assumptions, studies have reported that vestibular stimulation at the peripheral organ may temporarily increase the perceived length^9,10^ and width^9^ of the own hands or decrease the perceived width of own thighs^11^. These observations suggested that vestibular information indeed may be used to scale the internal representation of body segments. Accordingly, it could be expected that not only the perception of single body segments, such as hand or thigh, but also the perception of larger parts of one’s own body image is manipulated by vestibular input.

This latter prediction was recently investigated. Karnath *et al*.^12^ used an immersive virtual reality setup with biometric avatars to investigate the effects of vestibular stimulation on size estimation of whole extremities. Healthy subjects were asked to adjust their own arms and legs (that they perceived by seeing a virtual first person avatar) to the ‘correct’ length from various start lengths before, during, and after vestibular stimulation. Neither vestibular stimulation of the horizontal semicircular canal by caloric irrigation (CVS) nor of the whole vestibular nerve by galvanic stimulation over the mastoid (GVS) had a modulating effect on the estimated size of extremities. Subjects showed unaltered body size perception despite a clearly induced tonic imbalance in the vestibular system.

The straightforward explanation for this unexpected finding is that size perception of (large parts of) the body is not mediated by vestibular information. However, it is also possible that visual input plays a decisive, if not *the* decisive^13,14^, role for the perception of one’s own extremities. In that case, it is possible that the visual feedback on arms and legs may have overridden possibly existing modulations of body perception by vestibular stimulation in the study by Karnath *et al*.^12^. In fact, the previously reported effects of vestibular stimulation on hand size by Lopez *et al*.^9,10^ and on thigh width by Schönherr and May^11^ were based on tactile and/or proprioceptive information only; vision was excluded in these experiments. If visual feedback indeed plays this decisive role for the representation of our body image, modulations on the perceived size of arms and legs by vestibular stimulation should only be detected if vision of the extremities is excluded. The present study investigates this hypothesis. In contrast to the procedure used by Karnath *et al*.^12^, we altered the virtual reality (VR) scenario so that it only presented two blocks in 3D space on either the left or the right body side. Subjects were instructed to adjust the position of the blocks from different start positions until they had the feeling that they could just touch them with the tip of the left/right middle finger and with the left/right heel, respectively.

## Methods

### Participants

The number of participants was determined by a power analysis^12^: effect sizes were calculated based on previous data by Lopez *et al*.^9^ on mean perceived length of the own hand obtained in healthy subjects under CVS in contrast to sham stimulation. Assuming a minimum improvement of 0.9 cm, the corresponding effect size $d=\frac{0.9}{1.69}=0.53$ indicates a medium effect according to Cohen. For our power calculation, we therefore assumed a medium effect size of f = 0.25 for the body side (left, right) × condition (pre, stimulation, post) interaction, alpha = 0.05, power = 0.80^15^, and an assumed correlation of r = 0.65 between repeated measures using GPower 3.1 software^16^. This calculation yielded a required sample size of 20 participants. Our final sample consisted of 23 newly recruited, healthy subjects with a mean age of 24.17 ± 3.13 years (range 18–30 years; 7 male; 2 left-handed). They had no previous or current mental disorders, chronic diseases, vestibular disorders or motor impairments and provided informed consent. Two other participants were excluded; one due to extreme outlier values in all conditions, the other due to technical problems. All subjects provided written informed consent (i) to participate in the study and (ii) for publication of identifying information/images in an online open-access publication. The study was conducted in accordance with the ethical guidelines from the Declaration of Helsinki and was approved by the local ethics committee of the University Tübingen.

### Study design

**Figure 1:**
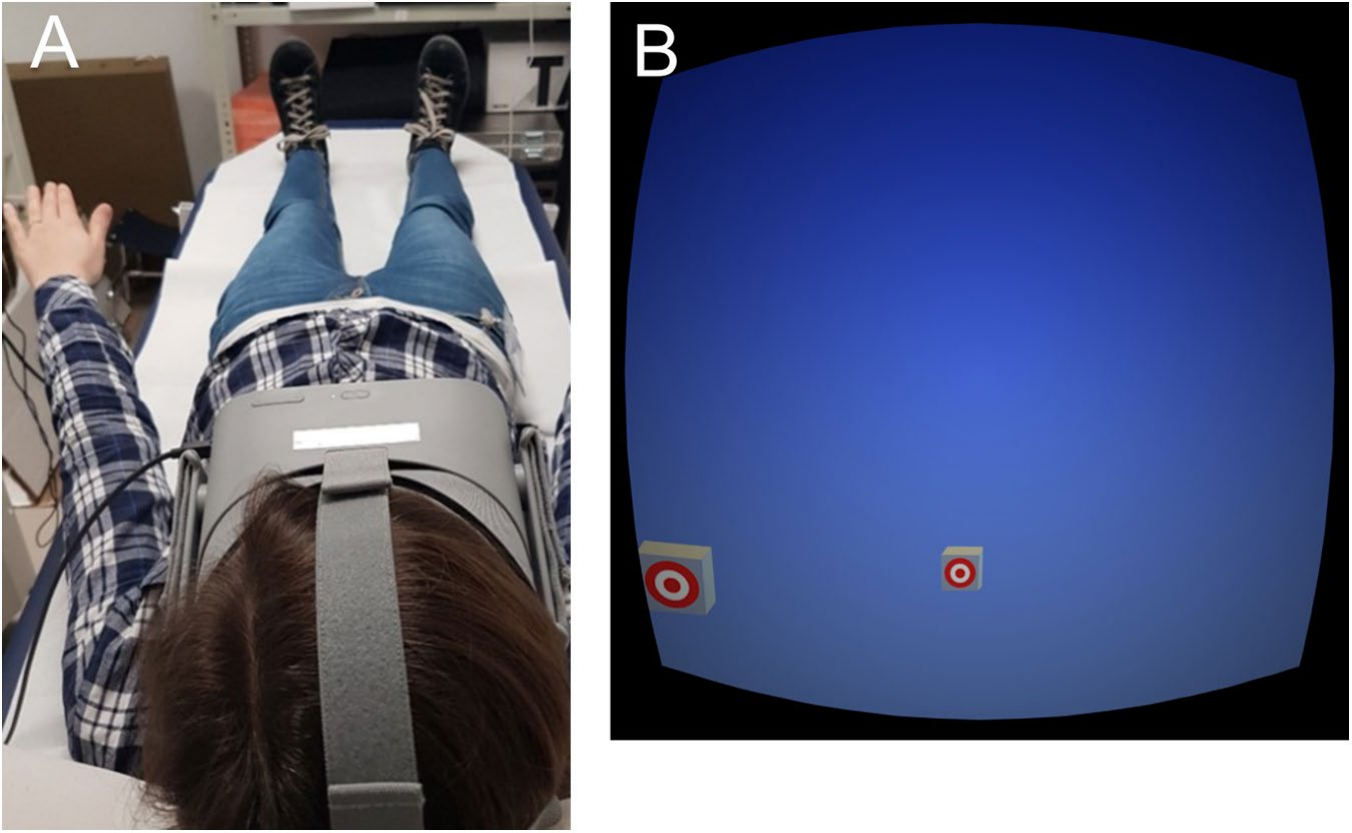
Study setup. Illustration of the study setting (**A**) and virtual reality setup (**B**).

At the beginning, the experimenter confirmed the inclusion criteria and explained the study procedure, then assessed self-reported height and weight, measured arm length, leg length, and interpupillary distance, as well as tested the ability to see three-dimensionally with parts of the Titmus test (Stereo Optical Co., Chicago, Illinois, USA). The measures of arm and leg length were taken at least twice to increase validity. The participants laid down on a medical examination couch in supine position, the head tilted ~30° forwards (Fig. 1A). In a virtual reality (VR) scenario, they saw two blocks (one further forward, one further back) presented either left or right on a blue background (Fig. 1B). The subjects were instructed that the block located further forward shall correspond with the position of the tip of the middle finger, while the block located further back shall correspond with the position of the heel. The participants had to adjust the position of the blocks from different start positions. This was done until they had the feeling that they could just touch them with the fingertip and with the heel, respectively.

The experiment was about 105 min long and analogous to the CVS experiment performed by Karnath *et al*.^12^. The participants experienced three conditions each with 20 trials, starting with the baseline condition before stimulation. The second condition was under caloric vestibular stimulation (CVS) of the left external auditory canal. The third condition started 15 min after the end of the second condition, i.e. at a time when the caloric nystagmus had long decayed, to ensure that the effects of the stimulation had stopped. Finally, the participants completed a short questionnaire on perceived vertigo, side effects such as nausea or other physical changes and strategies during the task.

CVS consisted of cold-water irrigation of the left external auditory canal with about 30 ml of ice water for about 1 min. Before irrigation, the experimenter inspected the left tympanic membrane with an otoscope. Directly after stimulation, eye movements were observed using Frenzel glasses. All participants showed a brisk nystagmus with the slow phase to the left side. Assessment procedures were started immediately after Frenzel glasses were put off and the VR head mounted display put on, i.e. ~15 sec after irrigation. Participants were asked to inform the experimenter as soon as the induced vertigo had ceased. Since the assessment procedure under CVS typically lasted longer than the vertigo, this condition provided trials in which subjects were under vertigo as well as trials after participants had reported that vertigo had ceased. Analogous to the CVS experiment performed by Karnath *et al*.^12^, only trials under vertigo were included in the data analysis.

### VR setup to measure perceived arm and leg lengths

The setup was presented through an Oculus Go head mounted display. Technically, we used a modification of the setup presented in Karnath *et al*.^12^ so that we were able to relate the virtual distances to a body that matched the participant in terms of height, weight, arm length, and leg length. Participants were in supine position and the two blocks of 10 cm size were presented either on the right or on the left side. They could choose with which block they wanted to start moving and indicated their decision to the experimenter by lifting either the arm or leg, so that its position was in line with the respective block they were currently adjusting. The start positions of the blocks varied in each trial between ± 40%, ± 35%, ± 30%, ± 25%, ± 20% of the person’s arm/leg length. Arm and leg length were both adjusted in one single trial; the order was up to the participant. Participants were told that starting positions of arm and leg blocks were random. Presentation of the each 10 trials on the left and on the right side was counterbalanced. As a result, there were 20 trials per condition (left/right), covering all start positions and sides, with ten repetitions per arm/leg (as in Karnath *et al*.^12^). The sequence was the same for each participant. The participants adjusted the position of a block by verbal instructions (“closer”, “further away”) to the experimenter who adjusted its position accordingly. Block movements were displayed to the participant by increasing and decreasing the size along the visual axes. The morphing step size was 2.5% of the recorded arm and leg length (as in Karnath *et al*.^12^). When they had the feeling that they could just touch the blocks with the fingertip of their middle finger (arm) or the heel (leg), the experimenter started the next trial. The experimenter gave no feedback on accuracy.

### Outcome parameters

The participant’s actual leg length was measured as the distance between the broadest point of the hips and heel in a standing position without shoes. Arm length was measured as the distance between the tuberculum majus and fingertip of the middle finger in T-pose. The experimenter recorded the measured distances. Estimated arm and leg length was derived from the VR setup analogously to Karnath *et al*.^12^. That is, the blocks were technically linked to an invisible body whose arm and leg lengths matched the participants’ individual bodies. We therefore derived estimated arm and leg length from the same physical landmarks (distance tuberculum majus to fingertip and distance hips to heel) as the actual arm and leg length. Body perception indices (BPI) for the arms and the legs were calculated with the following formula: BPI = (estimated size/actual size) × 100. Arm and leg length estimates were averaged for each side of the body because we did not assume a priori any differences between length perception of arms and legs. The resulting BPIs for the left and the right side of the body for the three conditions were used in the data analysis (procedure as in Karnath *et al*.^12^).

### Data analysis

A 3 × 2 ANOVA was conducted on BPIs with side (left, right) and condition (pre, stimulation, post) as within subject factors. For the second condition, only trials under vertigo were included. This resulted in 6.3 ± 2.2 (range 3–10) trials instead of 20 and led to more short start positions on the right and more long start positions on the left. We further evaluated non-significant effects of condition by comparing the baseline condition with the stimulation using equivalence tests. The smallest effect size of interest (SESOI) was defined as the effect size we had 80% power to detect (as in Karnath *et al*.^12^). The tests were conducted in R with the TOSTpaired function of the TOSTER package^17,18^.

## Results

The average weight of the participants was M = 68.6 kg ± 15.0 kg, the average height was M = 1.73 m ± 0.08 m. The average arm length was M = 0.72 m ± 0.04 m, the average leg length was M = 0.85 m ± 0.04 m. In the baseline (pre stimulation) condition, participants overestimated their arm and leg lengths (left arm: + 19.0% ± 20.1; right arm: + 19.1% ± 22.7; left leg: + 16.6% ± 22.5; right leg: + 15.2% ± 24.6). Pairwise t-tests showed that overestimation did not differ between both sides of the body (t(22) = 0.66, p = 0.513). Overestimation did not differ for arms and for legs (right side: t(22) = 1.51, p = 0.145; left side: t(22) = 0.95, p = 0.351), neither. Average time to complete a trial was 32 ± 20 seconds. More specifically, it was 38 ± 19 seconds in the first condition, 40 ± 40 seconds under vestibular stimulation, and decreased to 26 ± 10 seconds for the non vertigo trials of the second block and 27 ± 12 seconds for the third condition.

All participants experienced vertigo after CVS application with an average strength of 7.13 ± 1.69 on a rating scale from 0 (no vertigo) to 10 (very strong vertigo). The vertigo lasted on average for 2.59 ± 0.55 min. Fifteen participants (65%) were stating nausea. Three participants reported other changes in their body (13%), such as e.g. sweating (further details see Table 1). As strategies, participants reported for examples, comparisons between the estimated leg and arm length, visualization of legs and arms in the scenario, and the attempt to grab/kick the blocks (Table 1).

**Table 1:** Questions and answers from the questionnaire given to the 23 subjects.

Did you feel vertigo?	Yes (N = 23), No (N = 0)
How strong was the vertigo on a scale of 0 to 10 (0 = no vertigo, 10 = very strong vertigo)?	M = 7.1, SD = 1.7
Did you feel nausea?	Yes (N = 15), No (N = 8)
Have you noticed any other changes in your body [besides vertigo] as a result of the stimulation?	Sweating (N = 1), field of vision rotated (N = 1), uncoordinated movements of arms and legs (N = 1)
Did you feel any effects of the stimulation after the break?	Mild headache (N = 3), mild vertigo (N = 1), nausea (N = 5), slight discomfort (N = 1), feeling fitter (N = 1)
Which strategies did you use to solve the task?	Comparison between the estimated arm and leg length (N = 1), looking at arms and legs during the breaks (N = 1), body/gut feeling (N = 4), visualization/imagination of arms and legs/body in the scenario (N = 5), moving arms/legs (N = 2), attempt to grab/kick the blocks (N = 3), try to mask out vertigo (N = 1)

M = mean, SD = standard deviation.

**Figure 2:**
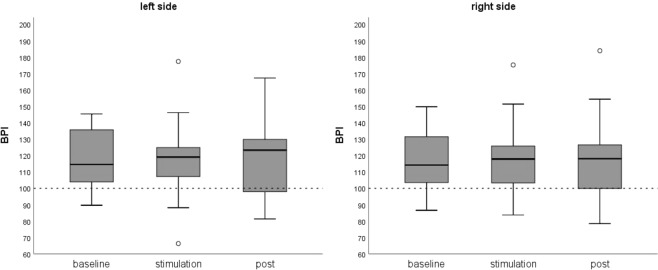
Body perception results. Boxplots of aggregated body perception indices (BPI; estimated/actual length * 100) for the left and right body side at baseline, under vestibular stimulation and at post-assessment. BPI > 100 indicates overestimation, BPI < 100 indicates underestimation. There was no significant change in BPI due to vestibular stimulation (left caloric vestibular stimulation with cold water; CVS).

Figure 2 and Table 2 show the BPIs of the participants for the left and right body side and all conditions. The 3 × 2 ANOVA on BPIs with body side (left, right) and condition (pre, stimulation, post) as within subject factors showed no significant main effect of side (F(1, 22) = 0.51, p = 0.481), no significant main effect for condition (F(2, 21) = 0.01, p = 0.994), and no significant interaction effect side*condition (F(2, 21) = 2.21, p = 0.122). This is also supported by equivalence tests comparing the first with the second condition. The equivalence tests for both sides were significant, suggesting equivalent estimates in the baseline condition and under CSV (left side: t(22) = −2.63, p = 0.008; alpha = 0.05; right side: t(22) = 2.56, p = 0.009; alpha = 0.05; equivalence bounds ± 0.61).

**Table 2:** Means (M) and standard deviations (SD) for the body perception index (BPI; (estimated/actual size) × 100) in each condition.

	BPI left		BPI right	
	M	SD	M	SD
(*N* = *23 subjects*)				
Pre	117.78	17.66	117.14	18.72
Stimulation	117.07	21.04	117.93	19.73
Post	118.61	22.86	116.81	24.39

BPI > 100 represents overestimation, BPI < 100 represents underestimation.

## Discussion

It had been discussed that the negative findings on a possible effect of vestibular signals on size perception of own extremities might have been provoked by the availability of visual feedback from the subjects’ arms and legs − though not from their real but virtual arms/legs. The present study followed the general procedure of the VR experiment by Karnath *et al*.^12^, but this time no visual information neither of the subject’s real arm/leg nor an arm/leg of a first person biometric avatar was provided. Subjects only saw two blocks in an otherwise empty space. However, despite a clearly induced tonic imbalance in the vestibular system, the newly recruited group of subjects again showed unaltered perception of arm and of leg size. Adjusted arm and leg lengths remained unchanged by stimulation, while over time, there was a descriptive, unspecific trend of increasing variability and speed in completing trials. In combination with the previous results^12^, our findings allow the conclusion that vestibular information does not serve to scale the internal representation of (large parts of) our body.

Along the assumptions on the role of vestibular input on body perception^5–8^ and the empirical observations on alterations of perceived size of hand and thigh^9–11^, this conclusion is surprising. However, the present as well as our earlier work^12^ represent two independent experiments with two independent groups of subjects, leading to the same conclusion. Our observations are also supported by previous work by Ferrè *et al*.^19^ who showed that perceived length and width of the hand was not influenced by vestibular stimulation. In this latter experiment, healthy subjects used a stick to indicate with one hand the perceived location of verbally identified landmarks on the other, occluded hand. While the present study very clearly ruled out visual feedback of own extremities as the decisive factor that might prevent vestibular manipulation of size perception of large parts of one’s own body, other contributing factors are conceivable (for discussion see Karnath *et al*.^12^). Further studies are needed to investigate such possible influence. However, it may also be permitted to question at this point whether the general hypothesis that vestibular signals have an impact on our internal representation of the body’s metric properties needs to be downsized.

Brandt and coworkers^20^ have suggested a reciprocal relationship between the human vestibular and visual systems throughout the vestibulo-thalamo-cortical pathways. On this basis Lopez^8^ proposed that one of the main functions of vestibular signals could be to link our body to the higher-order concept of the “self” and, in that context, modulate higher-order body representations, such as the perceived shape and size of the body. A conceptual problem of this idea is that the benefit of such a mechanism remains unclear. Wouldn’t it be expected, on the contrary, that negative effects would tend to result from such a link? If any head turn or other body movements – which represent the natural stimuli for our vestibular system – would indeed induce changes of, e.g., perceived arm length, one could expect misreaches to occur if we aim to grasp an object while moving the head or body at the same time. Likewise, alterations of perceived leg length resulting from head/body movements should induce a risk of stumbling or other instabilities while walking, climbing, etc. Thus, although it is obvious that vestibular signals are integrated with body representations, it seems that this happens in different contexts or at different levels of representation.

For example, it is quite conceivable that vestibular signals could be involved in coding of somatosensory detection, i.e. lower-order body representations. Ferrè and coworkers^21^ have observed increased somatosensory perceptual sensitivity as well as increased threshold for detecting pain^22^ immediately after left caloric vestibular stimulation (CVS), both ipsilaterally and contralaterally. In a further study, Ferrè *et al*.^23^ reported evidence not only for separate but also combined vestibular and visual modulation of somatosensation. One could well imagine that vestibular and somatosensory signals interact in a way that the vestibular signal, e.g., modulates the gain and/or synaptic connections of signal processing in the somatosensory pathway(s), converges with somatosensory signals on the same higher-order neurons, and/or changes connection weights between brain areas involved^21,24,25^.

Also, there is no doubt that vestibular signals serve our perception about the spatial relation of our body midline with respect to the world. Based on neurophysiological studies in monkeys as well as fMRI and psychophysical results in humans, it has been suggested that the brain uses internal maps of our surroundings that code topographical positions in head- and trunk-centered as well as world-centered frames of reference, rather than the retinotopic positions^26–37^. The coordinate transformation into these body-related internal maps is based on the afferent input from the retina, neck muscle spindles, and vestibular organs (cf. Figs 5 and 6 in ref.^38^) and is cortically represented in the so-called ‘multisensory (vestibular) cortex in humans’^3,4^. Accordingly, artificial vestibular or neck-proprioceptive stimulation in healthy subjects has been demonstrated to alter (under well control for possible effects of stimulation on motor pointing responses) our perception of own body orientation in space (‘subjective straight ahead’/‘body midline’)^39–41^. The results demonstrated that the input from the vestibular organs and neck muscles spindles contribute to computation of egocentric representations by affecting the internal representation of the body midline.

A decisive site for these computations is the multisensory (vestibular) cortex in humans^3,4^. Damage of (parts of) the right hemispheric ‘multisensory (vestibular) cortex’ leads to spatial neglect^3,42^ − a spontaneous and sustained deviation of eyes and head toward the ipsilesional side. This rightward bias is in the same direction as the neglect patients’ perceived spatial egocenter, i.e. their perception of “straight ahead” orientation of body midline^39,43^. While artificial vestibular or neck-proprioceptive stimulation has been demonstrated to cause a lateral bias of perceived spatial egocenter in healthy subjects (see above), manipulation of afferent input from these peripheral sensory organs compensates the lateral bias of neglect patients vice versa (vestibular stimulation: Silberpfennig^44^, Rubens^45^/visual, optokinetic stimulation: Pizzamiglio *et al*.^46^/neck-proprioceptive stimulation: Karnath *et al*.^47^).

### Caveats and limitations

So far previous experiments that reported an effect of vestibular stimulation on perceived length and width of own hands and thighs^9–11^ were performed in the real rather than a virtual reality environment. Thus, it can not be excluded that the present findings are due to the fact that vestibular stimulation has an impact on real but not on virtual reality environments. To investigate this possibility, the present observations could be supplemented by testing for possible effects of vestibular input on the perception of large body parts of the subject’s real body.

A further limitation of the study might be the non-randomization of the conditions. However, we chose an A-B-A design for all subjects to control for a possible positive behavioral effect if one should occur.

In order to maximize the impact of vestibular input on the behavioral measure, the present study used CVS. Cold caloric vestibular stimulation is a highly effective type of vestibular stimulation causing nausea and vertigo (cf. Table 1). We chose this type of vestibular stimulation to make sure we capture a possible positive behavioral effect if one should occur. The negative side is that the effect of this very effective type of vestibular stimulation does not last very long (minutes). Consequently, we could only analyze about a third of the CVS trials, which resulted in an unequal number of trials between the conditions. However, the total number of analyzed trials remained reasonable for the chosen statistics.

## Conclusion

In conclusion, previous findings in monkeys and humans have demonstrated that the brain uses the input from different afferent channels, including vestibular signals, to elaborate internal representations of egocentric space. Integration of input from the retina, vestibular organs, and neck muscle spindles is used to represent our body midline with respect to the surroundings. In contrast, our present and previous^12^ results have revealed that vestibular signals have no influence on our internal representation of shape and size of (large parts of) our body. It seems as if the egocentric representation of external space that allows us to perceive and adjust the position of our body relative to the environment clearly distinguishes from those internal representations of our body that serve the perception of our body’s metric properties. These two qualia appear to belong to different systems of body representation in humans.

## Data Availability

The datasets generated and analyzed during the current study are not publicly available due to the data protection agreement of the Center of Neurology at Tübingen University (approved by the local ethics committee) signed by the participants. The agreement covers data storage for a duration of 10 years at the Center of Neurology at Tübingen University. They are available via the corresponding author as well as the local ethics committee (ethik.kommission@med.uni-tuebingen.de) on reasonable request.

## References

[1] Bonnier P (1905). L’aschématie. Rev. Neurol. (Paris).

[2] Schilder, P. *The image and appearance of the human body*. New York: International Univ. Press (1935).

[3] Karnath H-O, Dieterich M (2006). Spatial neglect − a vestibular disorder?. Brain.

[4] zu Eulenburg P, Caspers S, Roski C, Eickhoff SB (2012). Meta-analytical definition and functional connectivity of the human vestibular cortex. Neuroimage.

[5] Pfeiffer C, Serino A, Blanke O (2014). The vestibular system: a spatial reference for bodily self-consciousness. Front. Integrat. Neurosci..

[6] Ferrè ER, Haggard P (2015). Vestibular-somatosensory interactions: a mechanism in search of a function?. Multisens. Res..

[7] Ferrè ER, Haggard P (2016). The vestibular body: Vestibular contributions to bodily representations. Cogn. Neuropsych..

[8] Lopez C (2015). Making Sense of the body: the role of vestibular signals. Multisens. Res..

[9] Lopez C, Schreyer H-M, Preuss N, Mast FW (2012). Vestibular stimulation modifies the body schema. Neuropsychologia.

[10] Lopez C, Nakul E, Preuss N, Elzière M, Mast FW (2018). Distorted own-body representations in patients with dizziness and during caloric vestibular stimulation. J. Neurol..

[11] Schönherr A, May CA (2016). Influence of caloric vestibular stimulation on body experience in healthy humans. Front. Integr. Neurosci..

[12] Karnath H-O (2019). Visual perception of one’s own body under vestibular stimulation using biometric self-avatars in virtual reality. PLos One.

[13] Schwoebel J, Coslett HB (2005). Evidence for multiple, distinct representations of the human body. J. Cogn. Neurosci..

[14] Crollen V, Albouy G, Lepore F, Collignon O (2017). How visual experience impacts the internal and external spatial mapping of sensorimotor functions. Scientific Reports.

[15] McDonald, J. H. *Handbook of Biological Statistics*. 3rd ed. Baltimore, Maryland: Sparky House Publishing (2014).

[16] Faul F, Erdfelder E, Lang A-G, Buchner A (2007). G*Power 3: a flexible statistical power analysis program for the social, behavioral, and biomedical sciences. Behav. Res. Methods.

[17] Lakens D (2017). Equivalence tests: a practical primer for t tests, correlations, and meta-analyses. Soc Psychol. Personal. Sci..

[18] Lakens D, Scheel AM, Isager PM (2018). Equivalence testing for psychological research: a tutorial. Adv. Methods Pract. Psychol. Sci..

[19] Ferrè ER, Vagnoni E, Haggard P (2013). Vestibular contributions to bodily awareness. Neuropsychologia.

[20] Brandt T, Bartenstein P, Janek A, Dieterich M (1998). Reciprocal inhibitory visual-vestibular interaction: visual motion stimulation deactivates the parieto-insular vestibular cortex. Brain.

[21] Ferrè ER, Bottini G, Haggard P (2011). Vestibular modulation of somatosensory perception. Eur. J. Neurosci..

[22] Ferrè ER, Bottini G, Iannetti GD, Haggard P (2013). The balance of feelings: vestibular modulation of bodily sensations. Cortex.

[23] Ferrè ER, Walther LE, Haggard P (2015). Multisensory interactions between vestibular, visual and somatosensory signals. Plos One.

[24] Beauchamp MS, Pasalar S, Ro T (2010). Neural substrates of reliability-weighted visual-tactile multisensory integration. Front. Syst. Neurosci..

[25] Haggard P, Iannetti GD, Longo MR (2013). Spatial sensory organization and body representation in pain perception. Curr. Biol..

[26] Andersen RA, Snyder LH, Li C-S, Stricanne B (1993). Coordinate transformations in the representation of spatial information. Curr. Opin. Neurobiol..

[27] Andersen RA, Lawrence H, Snyder LH, Bradshaw JA (1997). Multimodal representation of space in the posterior parietal cortex and its use in planning movements. Annu. Rev. Neurosci..

[28] Battaglini, P.-P., Galletti, C. & Fattori, P. Neuronal coding of visual space in the posterior parietal cortex. In: Their, P. & Karnath, H.-O., editors. *Parietal lobe contributions to orientation in 3D space*. Heidelberg: Springer, p. 539–553 (1997).

[29] Galletti C, Battaglini PP, Fattori P (1993). Parietal neurons encoding spatial locations in craniotopic coordinates. Exp. Brain Res..

[30] Brotchie PR, Andersen RA, Snyder LH, Goodman SJ (1995). Head position signals used by parietal neurons to encode locations of visual stimuli. Nature.

[31] Snyder LH, Grieve KL, Brotchie P, Andersen RA (1998). Separate body- and world-referenced representations of visual space in parietal cortex. Nature.

[32] Bottini G (2001). Cerebral representations for egocentric space: functional-anatomical evidence from caloric vestibular stimulation and neck vibration. Brain.

[33] Maravita A, Spence C, Driver J (2003). Multisensory integration and the body schema: close to hand and within reach. Curr. Biol..

[34] Chen Q, Weidner R, Weiss PH, Marshall JC, Fink GR (2012). Neural interaction between spatial domain and spatial reference frame in parietal-occipital junction. J. Cogn. Neurosci..

[35] Frankenstein J, Mohler BJ, Bülthoff HH, Meilinger T (2012). Is the map in our head oriented north?. Psychol. Sci..

[36] Schindler A, Bartels A (2013). Parietal cortex codes for egocentric space beyond the field of view. Curr. Biol..

[37] Chen Y (2014). Allocentric versus egocentric representation of remembered reach targets in human cortex. J. Neurosci..

[38] Karnath H-O (2015). Spatial attention systems in spatial neglect. Neuropsychologia.

[39] Karnath H-O (1994). Subjective body orientation in neglect and the interactive contribution of neck muscle proprioception and vestibular stimulation. Brain.

[40] Karnath H-O, Fetter M, Dichgans J (1996). Ocular exploration of space as a function of neck proprioceptive and vestibular input-observations in normal subjects and patients with spatial neglect after parietal lesions. Exp. Brain Res..

[41] Abekawa N, Ferrè ER, Gallagher M, Gomi H, Haggard P (2018). Disentangling the visual, motor and representational effects of vestibular input. Cortex.

[42] Karnath H-O, Rorden C (2012). The anatomy of spatial neglect. Neuropsychologia.

[43] Kapoor, N., Ciuffreda, K. J. & Suchoff, I. B. Egocentric localization in patients with visual neglect. In Suchoff, I. B., Ciuffreda, K. J. & Kapoor, N. (Eds), *Visual and vestibular consequences of acquired brain injury* (pp. 131–144). Santa Ana, CA: Optometric Extension Program Foundation (2001).

[44] Silberpfennig J (1941). Contributions to the problem of eye movements. III. Disturbances of ocular movements with pseudohemianopsia in frontal lobe tumors. Confin. Neurol..

[45] Rubens AB (1985). Caloric stimulation and unilateral visual neglect. Neurology.

[46] Pizzamiglio L, Frasca R, Guariglia C, Incoccia C, Antonucci G (1990). Effect of optokinetic stimulation in patients with visual neglect. Cortex.

[47] Karnath H-O, Christ K, Hartje W (1993). Decrease of contralateral neglect by neck muscle vibration and spatial orientation of trunk midline. Brain.

